# Emerging Role of Extracellular Vesicles and Cellular Communication in Metastasis

**DOI:** 10.3390/cells10123429

**Published:** 2021-12-06

**Authors:** Aisling Forder, Chi-Yun Hsing, Jessica Trejo Vazquez, Cathie Garnis

**Affiliations:** 1 Department of Integrative Oncology, British Cancer Research Center, Vancouver, BC V5Z 1L3, Canada; aforder@bccrc.ca (A.F.); cyh36@cam.ac.uk (C.-Y.H.); jtrejo@bccrc.ca (J.T.V.); 2 Division of Otolaryngology, Department of Surgery, University of British Columbia, Vancouver, BC V5Z 1M9, Canada

**Keywords:** extracellular vesicles, cancer, metastasis, tumor microenvironment, cellular communication

## Abstract

Communication between cancer cells and the surrounding stromal cells of the tumor microenvironment (TME) plays a key role in promoting metastasis, which is the major cause of cancer death. Small membrane-bound particles called extracellular vesicles (EVs) are released from both cancer and stromal cells and have a key role in mediating this communication through transport of cargo such as various RNA species (mRNA, miRNA, lncRNA), proteins, and lipids. Tumor-secreted EVs have been observed to induce a pro-tumorigenic phenotype in non-malignant cells of the stroma, including fibroblasts, endothelial cells, and local immune cells. These cancer-associated cells then drive metastasis by mechanisms such as increasing the invasiveness of cancer cells, facilitating angiogenesis, and promoting the formation of the pre-metastatic niche. This review will cover the role of EV-mediated signaling in the TME during metastasis and highlight the therapeutic potential of targeting these pathways to develop biomarkers and novel treatment strategies.

## 1. Introduction

An important prognostic determinant for cancer is the development of secondary metastatic tumors that arise in a separate location from the original primary lesion [1]. Following remission of a primary tumor, metastatic cancer may take years or decades to develop and is extremely difficult to treat. Cancer is the second leading cause of death in the world, and metastasis is responsible for 90% of cancer-related mortality [2]. In particular, lymph node metastasis is correlated with a significant decrease in the survival rate of cancer patients due to the potential for dissemination to multiple distant sites [3]. In contrast to the successful development of novel therapies for primary cancer, little progress has been made in treating metastasis. This is attributable to a lack of understanding of the biological mechanisms that underpin metastatic disease [4], which highlights the need for further research to identify the cellular and molecular factors that govern its development.

The process of metastasis, also known as the metastatic cascade, involves multiple steps: a local invasion of tumor cells from the primary tumor to surrounding tissues, intravasation into the circulatory system, arrest of the tumor cell at a site along the endothelium of the blood vessel, and extravasation into the surrounding tissue. At this time, a new secondary tumor can begin to grow within the permissive environment of the pre-metastatic niche (PMN) and finally progress into a new tumor [5,6]. Key processes involved in the steps of this cascade include epithelial–mesenchymal transition (EMT), which facilitates invasion of tumor cells from the primary tumor into surrounding tissues through a transformation of tumor cells to a migratory phenotype; angiogenesis, in which the formation of new blood vessels supplies nutrients to the primary tumor, as well as allowing intravasation and transport of the cancer cells to a distant site; and formation of the pre-metastatic niche which has supportive conditions for cancer cell growth at the site of future metastatic colonization [4]. These processes are not specific to a particular cell or cellular function but are rather a coordinated series of events requiring collective efforts and reciprocal signaling from the surrounding, dynamic tumor microenvironment (TME).

The TME is the area that encircles the tumor and contains both non-cellular and cellular components that can promote tumor progression and metastasis, with increasing evidence showing their involvement in drug resistance [7]. The extracellular matrix (ECM) makes up the non-cellular component and primarily consists of water, proteins, and polysaccharides, with the exact composition unique to the tissue type. The cellular aspect is composed of endothelial cells of the blood and lymph vessel networks, immune cells, fibroblasts, neuroendocrine cells, adipose cells, and additional factors [8]. Tumor cells and non-tumor components of the TME communicate bi-directionally to facilitate the invasion-metastasis cascade, as well as the formation and maintenance of the tumor microenvironment [7]. Complex networks of direct cell–cell contact and the release of soluble factors make up the different channels of intercellular communication [9]. These molecules are traditionally thought of as inflammatory regulators, growth factors, ECM remodeling enzymes, cytokines, and chemokines. Advances in research methods are revealing new mechanisms of communication that aid in the understanding of tumor biology. For instance, circulating tumor cells and cell-free circulating tumor DNA are shed from the solid tumor into circulation [8,10]. An emerging channel of intercellular communication is the trafficking of extracellular vesicles (EVs). 

**Figure 1:**
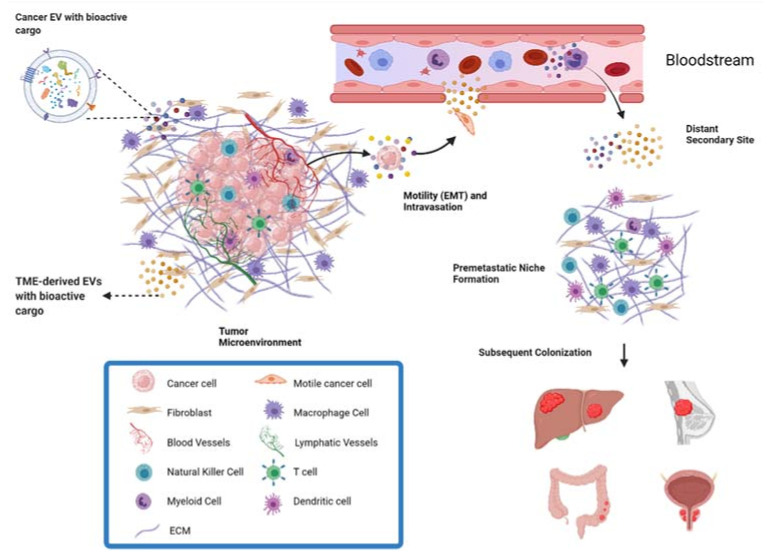
Overview of metastasis and contribution of EVs. EVs derived from both cancer cells and the cells of the TME can facilitate metastasis. EVs can act on surrounding cells in the TME to exert pro-metastatic effects, or act in an endocrine manner through the bloodstream. For example, EVs from cells of the TME can increase the motility and invasive capacity of cancer cells, allowing them to invade local tissue and intravasate into the bloodstream for transport to distant parts of the body. Through the endocrine pathway, EVs also play a role in preparing the premetastatic niche in a distant secondary site to be a supportive environment for metastatic cancer cells.

EVs are small, spherical, membrane-bound particles that are secreted into the extracellular space and deliver biologically active molecules to both nearby and distant sites [11]. EVs encompass both exosomes, which originate from the endosomal system of the cell, and microvesicles, which bud directly off the plasma membrane [12]. Cellular communication via EVs is known to modify the composition of the TME through diverse cargo that includes proteins, DNA fragments, mRNA, small RNAs (including microRNAs [miRNAs]), long noncoding RNAs (lncRNAs), circular RNAs (circRNA), lipids, and metabolites [13,14]. Receptors and ligands on the surface of the EV facilitate specific targeting for biodistribution and can trigger signaling changes within the recipient cell. Alternatively, vesicle contents are released within the recipient cell after endocytic uptake of the EVs [13]. The mode of communication can be either paracrine, targeting cells of different types in the same region, or endocrine, targeting different cell types in different anatomical locations [15]. Thus, EVs play an essential role in both primary tumor growth and metastatic evolution (Figure 1). As a result, targeting the reciprocal communication between tumor cells and TME is a promising field for the development of new therapeutics [9].

Herein we describe the EV-mediated cell-cell communication between tumor cells and various cells of the tumor microenvironment and detail how this impacts metastatic processes.

## 2. Fibroblasts

**Figure 2:**
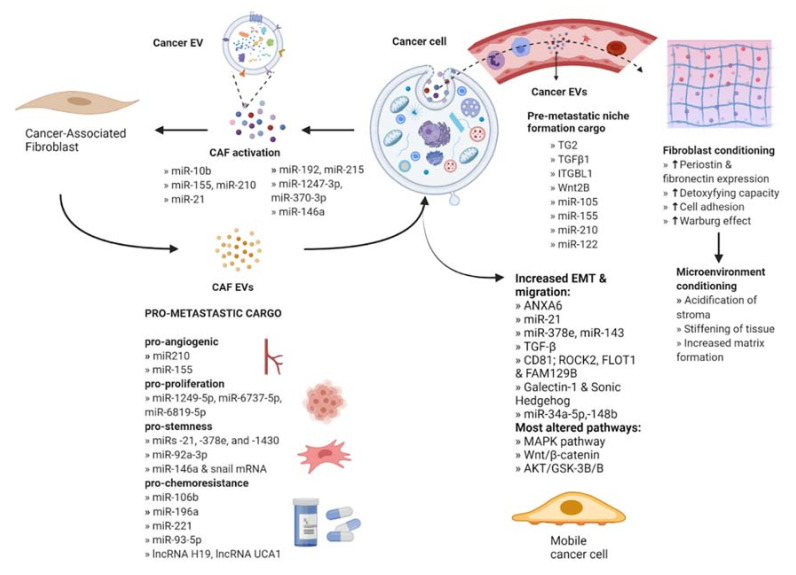
Intercellular communication between cancer cells and stromal fibroblasts. Cancer-derived EVs act on fibroblasts to facilitate activation into cancer associated fibroblasts (CAFs). CAF EVs act on cancer cells to enhance their metastatic potential by delivering bioactive molecules. The released cargo modifies several key signaling pathways that promote angiogenesis, proliferation, stemness, chemoresistance, and EMT. Cancer EVs also act at a secondary site via the bloodstream through activation of local fibroblasts to CAFs which condition the microenvironment for the establishment of the pre-metastatic niche.

Fibroblasts are the most abundant type of stromal cells and function to mediate tissue repair as well as maintain normal tissue homeostasis by producing and reorganizing different ECM proteins [16]. They remain dormant and non-proliferative in normal tissues and undergo an explosive expansion during the early phases of wound healing followed by massive apoptosis once the wound is repaired. Under tumorigenic conditions, fibroblasts are irreversibly activated and transition into cancer-associated fibroblasts (CAFs) that display more migratory behavior and vulnerability to epigenetic modifications [17]. Furthermore, CAF-derived EVs contain a variety of bioactive cargo that has been associated with regulating tumor development, metastasis, and therapeutic resistance [18] (Figure 2).

### 2.1. CAF Activation by Cancer-Secreted EVs

Fibroblasts have been shown to transform into CAFs in response to acquired cargo from EVs, particularly miRNAs. For example, one study reported that EVs derived from breast cancer cells contained miR-146a, which targets TXNIP, a metabolic gene that can act as a tumor suppressor in recipient fibroblasts. The miR-146a/TXNIP axis stimulated the Wnt/β-catenin signaling pathway, which mediated activation into CAFs [19]. Similarly, other studies have reported that the TGF-β, NF-κB, and PI3K/Akt/mTOR pathways were altered by EVs enriched with miR-192/miR-215, miR-1247-3p/miR-370-3p, and miR-10b respectively [20,21,22], and a CAF-like differentiation resulted. The activation of JAK2/STAT3 and PDK1/AKT signaling pathways by tumor-derived EVs containing miR-155/miR-210 or miR-21 has been shown to induce CAF activation; furthermore, activated CAFs showed increased expression of pro-angiogenic factors contributing to cancer cell migration [23,24,25]. In addition, certain oncogenic EV cargo, including miR-155 in pancreatic cancer, has been shown to target tumor suppressors like TP53 and TP53INP1 to promote CAF activation [21,26]. 

CAFs in the TME display high heterogeneity with distinct phenotypes, gene expression, and functions in tumor progression and subtypes have been observed to vary depending on cancer subtype and aggressiveness. There are two different types of CAF subpopulations, one exhibiting a myofibroblastic phenotype and the other exhibiting an inflammatory phenotype, also named myoCAFs and iCAFs. The myofibroblastic phenotypes are generally induced by treatment with TGF-β, while only a few miRNAs can induce the chemokine-expressing phenotype. Cargo in EVs, e.g., miR-155, miR-193b, and miR-210, can stimulate a CAF phenotype as indicated by induction of chemokines expression, some of which are linked to a worse prognosis [27]. Increased secretion of tumor-promoting factors, such as chemokines and miRNAs, are delivered from CAFs to target cells via EVs to further tumor initiation [28,29,30]. Distinct features in the different phenotypes include increased proliferation, ECM remodeling, and autocrine signaling ability [17,18], which may play a role in the link between CAF subtype and cancer prognosis.

### 2.2. The Role of CAF-Derived EVs in Metastasis

CAF EVs are known to stimulate metastasis by increasing the invasive capacity of cancer cells. An important step in metastasis is EMT, whereby cells lose their polarity as well as their adhesion to surrounding cells, changing to a spindle shape and gaining migratory potential. Bioactive cargo in CAF EVs can trigger this change; for instance, EV-mediated uptake of the protein ANXA6 has been reported to inhibit the repression of EMT signaling in pancreatic ductal adenocarcinoma. The protein forms a complex with LRP1/TSP1 in CAFs, and its EV-mediated delivery to cancer cells resulted in increased migration and invasion [31]. Conversely, direct interaction of CAF EV-derived cargo including miR-21, miR-378e, miR-143, or TGF-β with cancer cells has also been shown to promote EMT [32,33]. There is an enhancement in EMT and migration of cancer cells observed upon delivery of certain EV cargo such as CD81, ROCK2, FLOT1, and FAM129B, Galectin-1, and Sonic Hedgehog [34,35,36]. In fact, CAFs activated by cancer-derived EVs were observed to increase the invasive capacity of co-cultured cancer cells more than CAFs that were activated by cancer cells through direct interaction [37]. In addition, the core components of the planar cell polarity signaling pathway that controls tissue polarity have been shown to be essential for the EV-induced protrusive activity and motility of breast cancer cells [32,35]. 

Profiling the content of CAF-derived EVs has identified a role for RNAs in inhibiting tumor-suppressing pathways, thus promoting metastasis (Table 1). For example, CAF EV-encapsulated miR-181d-5p and miR-34a-5p downregulated the tumor-suppressor genes *HOXA5* and *AXL,* respectively, which is associated with inhibition of proliferation and mobility via the AKT/GSK-3β/β-catenin signaling pathway [38,39]. Similarly, miR-17-5p initiated a RUNX3/MYC/TGF-β1 positive feedback loop to confer an invasive phenotype [40]. In addition, long non-coding RNAs (lncRNAs) have been shown to regulate the expression of exosomal miRNA. By way of illustration, levels of LINC00355, a lncRNA that is associated with bladder cancer, were elevated in CAF-derived EVs as well as in the cells that were incubated with the EVs. The lncRNA decreased miR-15a-5p levels through a sponge mechanism, leading to the upregulation of HMGA2 that is associated with increased invasiveness in a variety of cancers [41,42]. Another example is the transfer of CAF-derived exosomes with high expression of LncRNA LINC00659 to colorectal cancer cells where this molecule sponges miR-342-3p, leading to the increase of its target ANXA2 [43]. This protein supports cancer progression by stimulating proliferation, migration, invasion and EMT progression. Furthermore, concentrations of the anti-tumoral miR-148b, miR-320a, miR-150-3p, and miR-335-5p were observed to be significantly decreased in CAF EVs, which corresponds to the activation of pro-tumor pathways and inducing EMT (Table 2) [31,44,45,46,47].

On rare occasions, CAF-derived EVs can suppress cancer progression and metastasis. One example in breast cancer is the shuttling of microRNA-1-3p, which inhibits GLIS1 and helps to counter invasion (Table 2) [48]. One finding with direct potential therapeutic value is that the inhibition of FAK tyrosine kinase expression within CAFs resulted in increased levels of tumor suppressors, miR-148a and miR-16, in secreted EVs. As a result, there was a decrease in breast cancer cell migration and metastasis [49].

### 2.3. Participation of CAFs in Pre-Metastatic Niche Formation

Cancer cells can also use EVs in an endocrine fashion, secreting them into the circulation to reach distant sites. This allows the preparation of a secondary site in the normal tissue called the pre-metastatic niche (PMN), which functions as a favorable environment for colonization by disseminated tumor cells. In particular, EVs activate fibroblasts at the site of the future PMN to carry out a variety of preparatory functions. Factors such as tissue transglutaminase 2 (TG2), a cross-linking enzyme, and TGFβ1, when transferred by cancer EVs to distant fibroblasts, trigger the production of matrix proteins, periostin, and fibronectin to alter the ECM for successful metastasis [50,51,52,53]. TG2 and cross-linked fibronectin have been observed to be upregulated in EVs derived from metastatic breast cancer cells and regulate the expression of various pro-metastatic proteins in recipient fibroblasts [50,54]. Additionally, EVs from primary colorectal cancer cells enriched with ITGBL1 or Wnt2B, have been shown to stimulate NF-κB signaling pathway or Wnt/β-catenin signaling, respectively, to activate resident fibroblasts. The activated fibroblasts, in turn, released pro-inflammatory cytokines that have been previously associated with the formation of the pre-metastatic niche [55,56].

Additionally, cancer EVs elicit metabolic reprogramming in resident fibroblasts to prepare the PMN. One study demonstrated that melanoma-derived EVs carrying miR-155 and miR-210 reprogrammed fibroblast metabolism to condition the TME. The fibroblasts exhibited the Warburg effect, characterized by an increase in aerobic glycolysis and a decrease in oxidative phosphorylation, leading to acidification of the stroma that was observed to inhibit the local immune response by deactivating T lymphocytes [57]. Breast cancer EV-derived miR-105 has been shown to induce the oncogenic protein MYC and activate the MYC pathway in CAFs at the future PMN. This resulted in an increased ability to compensate for changing metabolic needs of cancer cells as well as increasing the CAF’s capacity to consume toxic metabolites such as ammonium that may interrupt cancer cell development [54].

The role of CAF-derived EVs, in comparison to cancer-derived EVs, in regulating distant stromal cells at the future PMN is not yet well studied in the literature. However, CAF EVs within salivary adenoid cystic carcinoma induced CAF activation in lung fibroblasts, leading to subsequent ECM remodeling via integrin α2β1 to promote PMN formation [58]. 

### 2.4. CAF EVs in Stemness and Drug Resistance

Cancer stem cells (CSCs) are a unique subpopulation of tumor cells that are thought to be produced by the dedifferentiation of somatic cells. CSCs have defining characteristics, such as indefinite proliferation and the ability to give rise to a specialized adult cell type [59]. Between periods of quiescence, they are capable of self-renewal and of producing non-stem cell progeny, thus they have the capacity to facilitate long-term tumor growth. They are also capable of migrating to distant sites as well as developing resistance to different chemical and physical stimuli [60]. These characteristics underlie the hypothesis that CSCs can drive persistent malignancies and recurrence after treatment, which is supported by the enrichment of CSC markers in several recurrent cancers [61].

Stemness can be promoted by CAF EVs in different ways. EVs released by CAFs transfer the protein Wnt to cancer cells, promoting de-differentiation into a CSC phenotype, or can transfer miR-92a-3p to promote Wnt signaling [61,62]. One study reported that miR-21, miR-378e, and miR-143 each increased stem cell markers and mammosphere formation in breast cancer cells, while miRNA-221 packaged into EVs from CAFs enhanced hormone therapy resistance in breast cancer cells leading to bone metastasis [63,64].

Additionally, EV-mediated transfer of cargo from CAFs has been shown to improve the survival of cancer cells and stimulate chemoresistance by sequestering stemness inhibitors within the tumor cells. CAFs were observed to be intrinsically resistant to gemcitabine and were shown to confer this resistance in non-small cell lung cancer [65]. Pancreatic cancer-derived EVs carrying miRNA-106b directly targeted TP53INP1, promoting gemcitabine resistance [66]. CAF EV-derived lncRNA H19 has been shown to activate the β-catenin pathway by acting as a sponge for miR-141, thus enhancing chemoresistance in CRC [67]. Another study reported that CAF EVs containing lncRNA UCA1 sponges miR-103a, which had been reported to inhibit cisplatin resistance and tumor growth in vulvar squamous cell carcinoma (VSCC) cells [68]. Low expression of stemness inhibitor miR-7641 is also seen in CAF EVs compared to normal fibroblasts, promoting stem cell characteristics in recipient cancer cells [69]. Another example, miR-93-5p, is transferred from CAFs by EVs and down-regulates FOXA1 in colorectal cancer, conferring radioresistance [70]. Lastly, miR-196a from CAF EVs was demonstrated to bind to CDKN1B and ING5 in head and neck cancer cells, resulting in cisplatin resistance [71].

### 2.5. Packaging Machinery for CAF EVs Contributes to Chemoresistance

In addition, molecules that enhance the packaging of pro-stemness miRNAs similar to those described above into CAF EVs contribute to resistance. One study reported that Pumilio2, an RNA binding protein, facilitated miR-103a-3p and miR-130a packaging. Transfer of these miRNAs to cancer cells promoted cisplatin resistance via inhibition of apoptosis [65,72]. Resistance to treatment could be reduced by blocking the release of EVs from CAFs exposed to chemotherapy. In pancreatic cancer, this blockade reduces chemoresistance by preventing the transfer of pro-stemness molecules like miR-146a and snail mRNA [73].

## 3. Endothelial Cells

**Figure 3:**
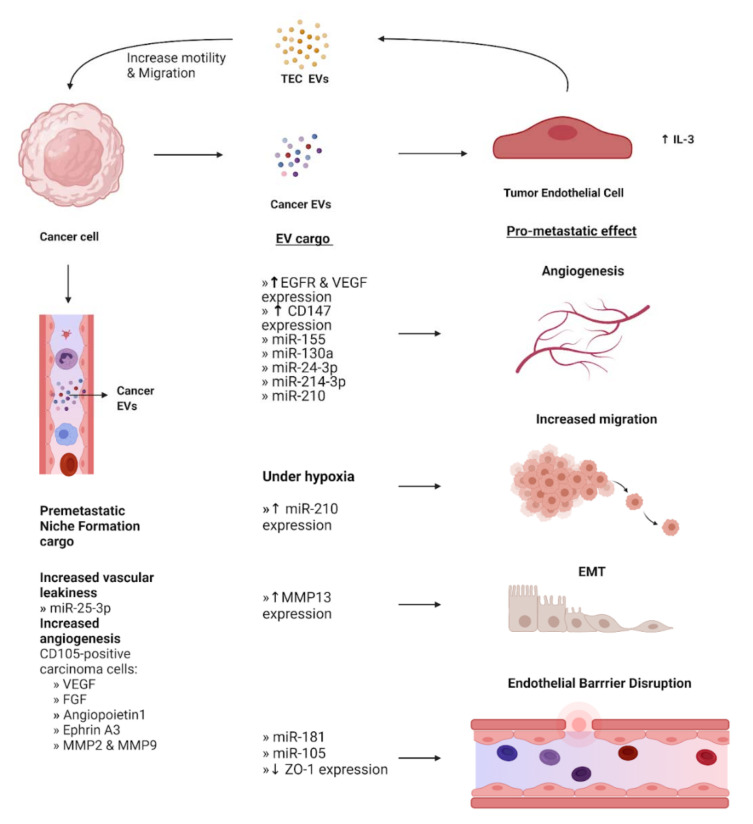
EV-Mediated crosstalk between cancer cells and endothelial cells. EVs containing cargo from cancer cells have various effects in the phenotype of surrounding endothelial cells, converting them into tumor endothelial cells (TECs). These effects include promoting angiogenesis, EMT, and increased migration under hypoxia. Additionally, cancer EVs mediate disruption of the endothelial barrier to aid the intravasation of cancer cells. EVs derived from TECs act on cancer cells to increase their motility and facilitate migration. Concurrently, cancer cells secrete EVs that travel to a secondary site and stimulate local endothelial cells that assist in the formation of the pre-metastatic niche. Expression levels of EV cargo are indicated by upward and downward arrows to represent increase and decrease, respectively.

The endothelium is a single layer of endothelial cells (EC) that lines the interior surface of all vessels in the cardiovascular and lymphatic systems and acts as an interface between the circulation and surrounding tissue. In non-cancer conditions, ECs and signals from ECs are crucial in the organization of the growth and development of connective tissues. ECs carry out myriads of tissue and organ-specific functions due to their capacity to adapt to their microenvironment [74]. They also elicit various responses from target cells by secreting EVs directly into the circulation. Interactions between EV surface proteins and the membrane receptors of the ECs then mediate EV uptake [75]. Phage display analysis has shown that ECs of the endothelium express various surface receptors depending on their location and functional status [76]. Therefore, ECs are important mediators of receptor-specific EV uptake in the endothelium and have been implicated in a variety of metastatic mechanisms (Figure 3).

### 3.1. Supporting the Formation of the Pre-Metastatic Niche

The formation of the PMN is characterized, in part, by angiogenesis to support migrating tumor cells, which can be mediated by EVs. In renal carcinoma, tumor-derived EVs contained elevated levels of multiple miRNAs including miR-19b, miR-29c, and miR-151 and significantly enhanced the expression of pro-PMN genes in lung endothelial cells, including the pro-angiogenic VEGFR1, VEGF, and MMP9 [77,78]. In colorectal cancer, cancer EVs containing miR-25-3p targeted vascular endothelial cells, inducing vascular permeability and angiogenesis which correlated with poorer patient prognosis. In addition, the researchers showed that exosomal miR-25-3p was shown to increase vascular leakiness and thus mediate the formation of the PMN in nude mice [79].

### 3.2. Disruption of Endothelial Barriers and Junctions

The metastatic potential of tumor cells is dependent on their ability to adhere to the endothelium and then disrupt the barrier, leading to transendothelial migration into the circulation [80]. Enhanced vascular permeability is critical for metastasis beyond tumor cell dissemination as it also enables the leakage of plasma proteins, and further exchange of EV and EV cargo at the secondary site [81,82]. Cancer-derived EVs have been shown to contain cargo that assists in achieving this vascular destabilization. In the brain, metastasis requires cancer cells to traverse through the blood–brain barrier that is formed by tight junctions between the cerebral endothelial cells. Brain cancer cells release EVs containing miR-181c that down-regulate the target gene *PDPK1* in ECs. This downregulation led to abnormal localization of the critical tight junction protein actin and cancer cell escape from the blood–brain barrier [81]. Furthermore, breast cancer cells secrete EVs containing miR-105 that is targeted to the endothelium and decreases ZO-1 protein expression to disrupt tight junctions both in vitro and in vivo. Treatment with anti-miR-105 in MDA-231-HM xenograft animal models reduced tumor volume and induced apoptosis in the tumor cells, suggesting an important role for miR-105 transported by EVs in the disruption of endothelial barriers in breast cancer [82].

### 3.3. Promotion of Angiogenesis by Endothelial Cells

Angiogenesis, the formation or sprouting of new blood vessels from pre-existing vessels, is co-opted in cancer to provide sufficient oxygen and nutrients to sustain tumor growth as well as enable dissemination of cancer cells to the circulation for metastasis [83]. While ECs tend to be quiescent, during angiogenesis, they proliferate rapidly, degrade the ECM, migrate towards angiogenic cues, and establish the tubular structures that form the basis of new blood vessels [84]. This process is regulated by the interactions between tumor cells, ECs, growth factors, and ECM components [85] in which EVs play an important mediating role. 

An EV cargo of interest in the promotion of angiogenesis is the activated protein epidermal growth factor receptor (EGFR), as it displays oncogenic properties in multiple malignancies. In endothelial cells, EGFR is selectively over-expressed in tumor-associated vasculature and is shed as cargo in EVs from cancer cells [86]. ECs incubated with EVs derived from alveolar, colorectal, and squamous cell carcinoma lines carrying oncogenic EGFR resulted in the intake and retention of these receptors, the expression of vascular endothelial growth factor, VEGF, and the activation of VEGF-receptor-2 [87]. VEGF, also referred to as VEGFA, has been extensively studied in the context of angiogenesis and lymphangiogenesis. In glioblastoma, cancer cell-derived EVs carrying high levels of VEGFA have been observed to induce pro-angiogenic and pro-permeability potential in brain endothelial cells. Circulating EVs collected from glioblastoma patients clinically confirmed the elevated measures of VEGFA [88]. The expression of VEGF in combination with enhanced degradation of the ECM via increased activity of matrix metalloproteinases (MMP) has been associated with increased tumor aggressiveness both in vivo and in vitro [89]. High expression of EVs containing CD147, an extracellular MMP inducer, has been observed in several cancers such as lung and colorectal carcinoma and CD147-positive EVs from epithelial ovarian cancer cells stimulated pro-angiogenic activity in ECs [90].

Several miRNAs in EV cargo have also been implicated in promoting angiogenesis. miR-155 transported in gastric cancer EVs caused a decrease in c-MYB and an increase in VEGF expression, in line with the promotion of vascular growth and metastasis [91,92]. Similarly, gastric cancer EVs containing miR-130a were delivered to ECs, which promoted angiogenesis through targeting c-MYB [93]. In breast cancer, it has been shown that miR-210 in EVs released by cancer cells is delivered to endothelial cells through the neutral sphingomyelinase 2 (nSMase2)-regulated secretory machinery and contributes via enhanced angiogenesis [94]. Clinically, the expression of miR-210 in breast cancer is correlated with a poorer prognosis and its expression is significantly higher in breast cancer patients with lymph node metastasis compared to those without [95]. In addition, endothelial cell-derived EVs play a role in promoting angiogenesis in triple-negative breast cancer. IL-3 activates tumor endothelial cells (TECs) and leads to altered miR-24-3p and miR-214-3p content in TEC-derived EVs, promoting metastasis [96,97].

Furthermore, a hypoxic state is a common feature in malignant tumors due to their intensive proliferation and subsequently increased oxygen demand. Hypoxia occurs when the oxygen demand is greater than the supply, resulting in an abnormally low level of oxygen tension. As a sufficient vascular supply is required for tumor growth, hypoxia is a major regulator of tumor metastasis and a driver of angiogenesis [98]. Hypoxia-induced EVs collected from both esophageal squamous cell carcinoma cells and leukemia cells contained increased miR-210. They promoted greater proliferation, migration, invasion, and enhanced tube formation in ECs in vitro and in vivo than the EVs cultured in normal conditions revealing an important role for miR-210 across multiple cancers [99,100]. 

### 3.4. Endothelial Stimulation of Epithelial Mesenchymal Transition

Epithelial to mesenchymal transition (EMT) increases the motility and invasive potential of cancer cells. EV-mediated, EMT-stimulating communication has been observed between nasopharyngeal carcinoma (NPC) cells and endothelial cells. MMP13 in EV cargo from NPC was found to up-regulate EMT in tumor cells by increasing the levels of MMP13 in surrounding human umbilical vein endothelial cells and stromal human skin fibroblast cells [101]. Additionally, EVs derived from doxorubicin-resistant human microvascular endothelial cells incubated with NPC cells substantially decreased the concentration of epithelial markers such as E-cadherin and vimentin, indicating EMT had occurred, and increased proliferation and migratory capacity [102]. 

## 4. Immune Cells

**Figure 4:**
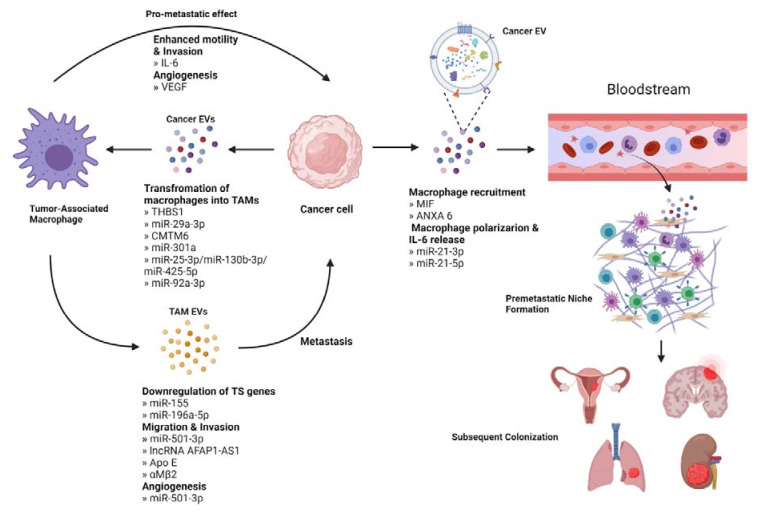
Interaction between cancer cells and tumor associated macrophage (TAMs). Cancer derived EVs deliver factors like miRNAs that promote the transformation of macrophages into TAMs. TAMs can stimulate metastasis directly or through EV-mediated downregulation of tumor suppressor genes, increased migration, and invasion potential, and supporting angiogenesis. Cancer cells also release EVs that act in an endocrine manner to stimulate the recruitment of macrophages in a secondary site. These macrophages then undergo polarization to support the establishment of the pre-metastatic niche, aid to evade the immune surveillance, and contribute to subsequent colonization.

Immune cells in the TME include macrophages, dendritic cells, lymphocytes, neutrophils, myeloid-derived suppressor cells, and natural killer cells [103]. Many of these cells play an EV-mediated role in primary tumor progression, for example, by assisting in the escape of the tumor from immunosurveillance [104]. Tumor-associated macrophages (TAMs) are the main immune cell type infiltrating the TME and have been shown to engage in bidirectional EV-mediated cellular communication with tumor cells to promote metastasis [105] (Figure 4).

### 4.1. Cancer EVs Educate TAMs to Promote Metastasis

EVs released from tumor cells can act on immune cells in the TME, commonly eliciting a pro-metastatic response through polarization of macrophages. Macrophages can be polarized to the classically pro-tumor M2 phenotype or the anti-tumor M1 phenotype, although studies in oral squamous cell carcinoma (OSCC) report a pro-tumor role for both M1 and M2 polarized macrophages in promoting metastasis. One study found that EVs containing THBS1 polarized macrophages towards the M1 phenotype, which when co-cultured resulted in increased motility of the tumor cells [106]. In contrast, OSCC EVs transporting miR-29a-3p and CMTM6 respectively transformed macrophages to the M2 phenotype and were associated with increased invasive potential [107,108]. In pancreatic cancer, hypoxic conditions trigger the release of EVs containing miR-301a, which then induce M2 polarization through the PTEN/PI3Kγ pathway. Co-culturing M2 TAMs resulted in increased motility and invasion of pancreatic cancer cells. It was shown that EMT had occurred, with a decrease in epithelial markers and an increase in mesenchymal markers [109]. Similarly, colorectal cancer-derived EVs containing a variety of miRNAs, including miR-25-3p, miR-130b-3p, and miR-425-5p were taken up by macrophages, which became polarized to the M2 phenotype through the PTEN/PI3K pathway and enhanced EMT [110]. These macrophages were found to secrete VEGF, which is associated with angiogenesis and thus tumor cell escape [111]. miR-25-3p, along with miR-92a-3p, is also released in liposarcoma-derived EVs and stimulated secretion of IL-6 from macrophages in vitro. When incubated with the conditioned media from these macrophages, increased migration and invasive potential of liposarcoma cells were observed [112].

### 4.2. TAM-Derived EVs Contribute to Metastasis

M2 TAMs can produce EVs that communicate with tumor cells to produce pro-metastatic effects. For example, in non-small-cell lung cancer, M2-derived EVs containing miR-155 and miR-196a-5p led to downregulation of RASSF4, a known tumor suppressor gene, and subsequently increased tumor cell migration, invasion, and EMT [113]. A miRNA-dependent EV communication method is also present in pancreatic adenocarcinoma, wherein M2 EVs containing miRNA-501-3p activate TGF-β in tumor cells to promote migration and invasion. Furthermore, these EVs caused increased expression of angiogenesis-related proteins VEGFA, VEGFR2, and ANG2 [114]. In esophageal cancer, EVs containing lncRNA down-regulated miR-261 in the tumor cells, leading to up-regulation of ATF2 and thereby promoting invasion, EMT and metastasis [115,116]. In a non-miRNA mediated pathway, M2 TAMs in gastric cancer transport ApoE in EVs to tumor cells, leading to PTEN/PI3K signaling and subsequent cytoskeletal remodeling to support migration [117]. Finally, in hepatocellular carcinoma, EVs derived from M2 TAMs transfer αMβ2 to the tumor cells to activate MMP-9. MMP-9 then induces degradation of the ECM to increase the migratory potential of the tumor cells [118].

### 4.3. The Role of EV-Mediated Tumor-Neutrophil Communication in Metastasis

Similar to macrophages, tumor-associated neutrophils are classified into distinct subpopulations: N1, which displays anti-tumor activity, and N2, which plays a role in cancer progression and metastasis [119]. The role of N2 neutrophils in metastasis includes developing the pre-metastatic niche, promoting angiogenesis and motility at the primary tumor site, and assisting in extravasation of circulating tumor cells [120]. A survey of the literature produced limited research into the possible role of EVs in this process. In a mouse model of lung metastasis, tumor-derived EVs containing small nuclear RNAs activated TLR3 in the alveolar epithelial cells, recruiting neutrophils to the pre-metastatic niche. Depletion of neutrophils in vivo prevented metastasis, suggesting a key role in the establishment of the pre-metastatic niche [121]. In gastric cancer, tumor-derived EVs induced N2 polarization of neutrophils in the TME through the NF-κB pathway, leading to increased tumor cell migration [122]. Despite the relevance of neutrophils in metastasis, further research is required to characterize the role of EVs in facilitating their effects.

### 4.4. Establishing the Immune Component of the Pre-Metastatic Niche

The first step of establishing the immune component of the PMN is the recruitment of macrophages to the future site of colonization by EV-mediated communication from tumor cells. In pancreatic cancer, which is known to metastasize to the liver, it has been shown that cancer-derived EVs containing macrophage inhibitory factor (MIF) bind selectively to Kupffer cells, specialized resident macrophages in the liver, causing the release of TGF-β. TGF-β then activates hepatic stellate cells, causing the up-regulation of fibronectin production and the subsequent recruitment of bone marrow-derived macrophages to the fibronectin-rich area of the liver to begin preparing the PMN [123]. In breast cancer, chemotherapy has been linked to lung metastasis. Chemotherapy causes the release of breast cancer-derived EVs containing ANXA6 that are targeted to the lung and activate the CCL2-CCR signaling axis, recruiting monocytes, which then differentiate into macrophages at this future site of metastasis [124].

Once macrophages have been recruited to the PMN, they undergo polarization in response to cancer-derived EVs and subsequently contribute to preparing a permissive environment for metastatic cancer cells. Ovarian cancer-derived EVs transport miR-21-3p to the site of the pre-metastatic niche, where it activates the STAT3 signaling pathway leading to M2 polarization of the local macrophage population. The same study also noted local immune suppression mediated by inhibition of T cells, NK cells, and dendritic cells in addition to increased production of IL-6 [125]. IL-6 is known to trigger feedback and cause further activation of the STAT3 pathway, reinforcing immune suppression by down-regulating pro-immune cells and up-regulating immune-suppressive cells like regulatory T cells and MDSCs while also promoting macrophage polarization [126]. Additionally, in colorectal cancer, tumor-derived EVs containing miR-21-5p bind TLR7 on Kupffer cells in the liver causing macrophage polarization and secretion of IL-6 [127]. This miRNA is the corresponding guide strand to miR-21-3p [128] transported by ovarian EVs.

## 5. Potential Applications of EVs in Clinical Management and Treatment 

The development of effective cancer treatments is hindered by tumor heterogeneity, and the complexity within the TME. Given the pivotal role of EVs in mediating intercellular communication, these factors represent a potential target for novel treatment approaches.

One application of cancer-derived EVs is as a biomarker for earlier diagnosis or treatment response predictions (Table 3 and Table 4). In particular, blood-based EVs and their miRNA cargo, which can be acquired and analyzed by minimally invasive techniques, have garnered considerable attention. For instance, EV integrins extracted from blood plasma have been shown to predict organ-specific metastasis from patients with breast and pancreatic cancer [129]. Additionally, though there has been an increase in overall survival for cohorts of metastatic colorectal cancer patients receiving targeted therapies, only a subset of patients respond favorably to such treatment. Recent expression analysis for a panel of miRNA species encapsulated in EVs successfully predicted the prognosis of metastatic CRC patients following first-line treatment with anti-angiogenic therapy [130]. EV-derived miR-21, a miRNA previously established with an oncogenic role in several cancers, from plasma has been also reported as a potential marker for recurrence in CRC. Its expression level was also significantly associated with the presence of liver metastases [131]. Furthermore, another study examined miRNA profiles of EVs secreted by primary and metastatic CRC cell lines that displayed different metastasis potentials. They revealed that exosomal miR-17-5p and miR-92a-3p expression were consistent with CRC progression [131]. Analysis of the two miRNAs on clinical serum samples showed that miR-17-5p was generally downregulated in CRC, but was expressed in higher levels from patients with distant metastases [131,132]. 

EVs have also been investigated as a carrier for nanotechnology-based drug delivery systems due to their delivery capabilities, including cell-specific targeting, internal stability for miRNA and proteins, and promotion of cargo transfer through endocytosis [133]. Using a rat model of glioblastoma, one study introduced EVs engineered to contain sponge-like constructs that inhibit miR-21. The engineered EVs resulted in a significant reduction in the volume of the tumors and prevented further metastasis, indicating an effective intervention [134]. In another example, triple-negative breast cancer cell lines were exposed to EVs with modified surface proteins targeting the Met onco-protein, which is regularly overexpressed in this subset of breast cancer. These engineered EVs were shown to improve cellular uptake efficiency and antitumor efficacy of chemotherapy medications—a finding that was validated in subsequent mouse model studies [135]. 

Finally, with further understanding of the importance of crosstalk between tumor cells and the TME, the biogenesis of EVs has also emerged as a target for the treatment of malignant tumors. Numerous inhibitors of EV release have been shown to prevent tumor progression and increase susceptibility to antitumor therapies [136]. Calpain inhibitors that modulate the release of intracellular calcium stores have been shown to block the calcium-dependent release of EVs in prostate cancer [137,138]. EV inhibition may also be mediated via treatment with MEK inhibitors, proton pump inhibitors, and regulating Rab protein concentrations [136,139]. A difficulty with blocking the release of EVs from tumors is controlling specificity; however, multiple studies have focused on targeting tumor-specific enzyme isoforms involved in different steps of tumor-derived EV biogenesis. The enzymes, peptidyl arginine deiminase 2 (PAD2) and 4 (PAD4), are overexpressed in malignant ovarian and prostate tumors. When inhibited by chloramidine, it was observed to minimize tumor-derived EV production, thus raising the sensitivity of the cells to chemotherapy [140]. 

Over the last decade, significant progress has been made in developing therapies that utilize EVs and the pathways they are involved in. One treatment in the first phase of clinical trials (NCT03608631) utilized engineered EVs, referred to as iExosomes, which are EVs derived from normal fibroblast-like mesenchymal cells. These EVs were engineered to carry shRNA that targets the oncogenic KRAS mutation G12D, and were found to suppress pancreatic cancer progression in multiple mouse models [141]. Another study modified luminescent porous silicon nanoparticles (PSiNPs) that are widely used as drug carriers, to be exocytosed by tumor cells in order to produce biocompatible exosome-biomimetic PSiNPs. They observed that the nanoparticles possessed strong cross-reactivity of cellular uptake and cytotoxicity against bulk cancer cells and cancer stem cells [142]. In addition, one group used tumor antigen (IFN-γ-)-loaded dendritic cell-derived EVs as maintenance immunotherapy after first-line chemotherapy for non-small-cell lung cancer [143].

Despite their specific roles in metastasis, targeting EVs for therapeutics presents numerous clinical challenges. In particular, EVs consist of a heterogeneous population in terms of origin, size, cargo, and surface-protein signatures as they are assembled and packaged in a cell-specific manner [144]. There have been studies with proteomic evidence suggesting a universal protein signature that is shared by most EVs, CD63 and CD9 for example, however, difficulties with isolating subpopulations and with identifying tumor-specific EV markers persist [145]. Other issues exist with controlling the clearance with EVs once administered. Similar to liposomes, the systemic introduction of EVs have been shown to lead to non-specific accumulation in the liver, spleen, gastrointestinal tract and lungs with homing differences related to EV cell origin [146]. In addition, EVs from allogeneic sources may induce undesired immune responses or other negative responses [144]. Each individual EV formulation may require careful evaluation of the immunogenicity and biocompatibility. The importance of EVs in promoting metastasis is undeniable, but further characterization will be required to fully recognize their therapeutic utility.

## 6. Conclusions

Extracellular vesicles, which are known to mediate intercellular communication within the TME, have been shown to play a role in promoting tumor progression and metastasis. Tumor cells secrete EVs that induce a pro-tumorigenic phenotype in non-malignant cells of the stroma, including fibroblasts, endothelial cells, and local immune cells such as macrophages. These tumor-educated cells then release EVs that contribute to the metastatic cascade by increasing the motility and invasive potential of tumor cells, facilitating angiogenesis and the formation of the pre-metastatic niche, and contributing to drug resistance among other mechanisms. Since EVs are a driver of metastasis, investigations into the molecular mechanisms that drive this crosstalk are a promising avenue for the development of future therapeutics. For instance, inhibiting the release or absorption of circulating EVs has been shown to decrease the incidence of metastasis in animal models [147]. EVs also have potential as a biomarker for primary tumors as well as recurrence and utility as a targeted drug-delivery system [148]. Further research is required to apply findings derived mainly from in vitro and animal models of cancer to the clinical setting, where targeting EV-driven transformation of the TME could be the key to preventing metastasis in patients.

## Figures and Tables

**Table 1:** Oncogenic cargo from extracellular vesicles of cancer-associated fibroblasts and their effect on cancer cells.

Author (Year)	Type of Cancer	Oncogenes Cargo in CAF EV	Target Tumor Suppressor	Effect
Wang H. et al., (2020)	Breast cancer	miR-181d-5p	HOXA5	Facilitates proliferation, invasion, migration and EMT.
Li Y. et al., (2018)	OSCC	miR-34a-5p	AXL	Increases proliferation and mobility by EMT.
Chen B. et al., (2021)	Breast cancer	miR-500a-5p	USP28	Modulates metastatic phenotype of cancer cells.
Zhang Y. et al., (2020)	Colorectal cancer	miR-17-5p	RUNX3	Confers an invasive phenotype.
Zhang Y. et al., (2021)	Bladder cancer	LINC00355	miR-15a-5p	Increases HMGA2 expression resulting in increased invasiveness.
Zhou L. et al., (2021)	Colorectal cancer	LINC00659	miR-342-3p	Promotes cancer cell progression.

**Table 2:** Loss of tumor suppressor cargo in extracellular vesicles derived from cancer-associated fibroblasts and their effect on cancer cells.

Author	Type of Cancer	Loss of TS in CAF EV	Target Oncogene	Effect
Tao S. et al., (2021)	Breast cancer	microRNA-1-3p	GLIS1	Increased cell viability, invasion, migration and EMT. Supports tumor formation and metastasis.
Li B. et al., (2018)	Endometrial cancer	miR-148b	DNMT1	Promotes cancer cell invasion and metastasis.
Zhang Z. et al., (2017)	Hepatocellular carcinoma	miR-320a	PBX3	Contributes to cell proliferation, migration and metastasis.
Yugawa K et al., (2021)	Hepatocellular carcinoma	miR-150-3p	-	Enables migration and invasiveness.
Wang F et al., (2017)	Hepatocellular carcinoma	miR-335	CDC 42, CDK2, EIF2C2, EIF5, LIMK1, NRG1, PLK2, and RGS19	Promotes cell proliferation and invasion.

**Table 3:** Completed clinical trials utilizing extracellular vesicles as biological markers and the description of the circumstances under which they are used. Information from ClinicalTrials.gov, accessed on 1 November 2021.

Study Type (Year) Estimated Enrollment	Study Title	Type of Cancer	Description
NCT03262311 Clinical Trial (2021) 21 participants	Pimo Study: Extracellular Vesicle-based Liquid Biopsy to Detect Hypoxia in Tumors	Invasive carcinomas: head and neck, lung, bladder, uterine cervix or breast	Hypoxia marker with prognostic and predictive value based on extracellular vesicles derived from blood samples to identify patients presenting tumor hypoxia that may benefit from sensitizer treatments or targeted radiotherapy.
NCT03228277 Clinical Trial Phase II (2017) 25 participants	Olmutinib Trial in T790M (+) NSCLC Patients Detected by Liquid Biopsy Using BALF Extracellular Vesicular DNA	Non-Small Cell Lung Cancer (NSCLC)	Assess the anti-tumor efficacy of Olmutinib (Olita^®^) administered to patients with T790M-positive NSCLC by extraction of DNA from extracellular vesicles of bronchoalveolar lavage fluid.
NCT02662621 Clinical Trial (2015) 71 participants	Pilot Study with the Aim to Quantify a Stress Protein in the Blood and in the Urine for the Monitoring and Early Diagnosis of Malignant Solid Tumors	Solid Tumors	Determine the utility of the stress protein HSP70, located at the membrane of EVs coming from cancer cells, as a marker for early diagnosis in blood and urine samples.
NCT04913545 Observational (2019) 18 participants	The Sensitivity and Specificity of Using Salivary miRNAs in Detection of Malignant Transformation of Oral Lesions	Oral Premalignant Lesions	Evaluate the diagnostic accuracy of salivary extracellular vesicles miRNAs to detect the malignant transformation of the premalignant lesion.

**Table 4:** Ongoing clinical trials registered in ClinicalTrials.gov (accessed on 1 November 2021) utilizing extracellular vesicles as biological indicators and the description of the circumstances under which they are implemented.

Study Type (Year) Estimated Enrollment	Study Title	Type of Cancer	Description
NCT04523389 Observational (2020) 172 participants	Contents of Circulating Extracellular Vesicles: Biomarkers in Colorectal Cancer Patients	Colorectal Cancer	Study the potential of miRNAs contained within exosomes derived from tumors as biomarkers of early prognosis from blood samples.
NCT04852653 Observational (2021) 40 participants	A Prospective Feasibility Study Evaluating Extracellular Vesicles Obtained by Liquid Biopsy for Neoadjuvant Treatment Response Assessment in Rectal Cancer	Rectal Cancer	Evaluate if the detection of tumor EVs from blood samples is a reliable biomarker for the differentiation of good responders to neoadjuvant chemoradiotherapy (nCRT). This will aid in the accurate identification of good responders to nCRT and spare them of the functional cost of total mesorectum excision.
NCT04742608 Observational (2020) 250 participants	Development of Liquid Biopsy Technologies for Noninvasive Cancer Diagnostics in Patients with Suspicious Thyroid Nodules or Thyroid Cancer	* Thyroid Gland Carcinoma * Thyroid Gland Nodule	Collection of blood and tissue samples from surgical resections of the thyroid. Posterior isolation and characterization of EVs, then perform an RNA and DNA panel to have a molecular profile to be used as a predictor for thyroid nodules or thyroid cancer.
NCT04164134 Observational (2018) 396 participants	New Strategies to Detect Cancers in Carriers of Mutations in RB1	Retinoblasto-ma (RB)	Development of non-invasive cancer test using blood samples for the detection of tumors through their derived EVs in RB1-mutation carriers, complemented with family cancer history.
NCT03957252 Observational (2019) 2800 participants	Validation of Clarity DX Prostate as a Reflex Test to Refine the Prediction of Clinically-significant Prostate Cancer	Prostate Cancer	Determine the accuracy of the blood test Clarity DX as a reflex to PSA by extracellular vesicle profiling on patients suspected of prostate cancer who will undergo biopsy. Results will be compared to assess predictive accuracy.
NCT04529915 Observational (2020) 470 participants	Multicenter Clinical Research for Early Diagnosis of Lung Cancer Using Blood Plasma Derived Exosome	Lung Cancer	Evaluate the possibility of distinguishing between normal and lung cancer patients through deep-learning analysis of blood abundant exosomes and the analysis of lung cancer specific exosomal protein.
NCT04638049 Interventional-Clinical Trial (2020) 50 participants	Intestinal Microbiota in Prostate Cancer Patients as a Biomarker for Radiation-Induced Toxicity	* Prostate Cancer * Prostate Adenocarcinoma * Prostatic Neoplasms	Examination of the microbiota composition (feces), the associated metabolome (blood, feces and urine) and bacterial extracellular vesicles (BEVs) (blood and feces) to establish a prospective biomarker in the pathophysiology of radiation-induced GI toxicity.
NCT04993378 Observational (2018) 40 participants	Prospectively Predict the Efficacy of Treatment of Gastrointestinal Tumors Based on Peripheral Multi-omics Liquid Biopsy	Advanced Gastric Adenocarcinoma	To verify that four plasma EV-derived proteins generate a signature score that robustly predicts immunotherapeutic outcomes during different stages of the disease.
NCT02514681 Interventional-Clinical Trial (2015) 370 participants	A Phase III Trial of Pertuzumab Retreatment in Previously Pertuzumab Treated Her2-Positive Advanced Breast Cancer	HER2-positive Locally Advanced or Metastatic Breast Cancer	Since Pertuzumab retreatment can be more effective than trastuzumab and chemotherapy-containing the study will evaluate its efficacy and safety. In addition, microRNA expression in extracellular vesicles after anti-HER2 therapy will be evaluated to find a prognostic and predictive biomarker.
NCT03576612 Interventional-Clinical Trial (2018) 36 participants	GMCI, Nivolumab, and Radiation Therapy in Treating Patients with Newly Diagnosed High-Grade Gliomas	Glioma, Malignant	Assessment of safety, maximum tolerated dose and toxicity of combining GMCI plus nivolumab with standard of care radiation therapy, and temozolomide to treat patients with newly diagnosed high-grade gliomas. Determination of immune biomarkers including serum extracellular vesicles (EVs) based on surface and content proteins.
NCT04581382 Interventional-Clinical Trial (2020) 20 participants	Radiation Therapy, Plasma Exchange, and Immunotherapy (Pembrolizumab or Nivolumab) for the Treatment of Melanoma	Melanoma	Establish the performance of radiation therapy, plasma exchange, and pembrolizumab or nivolumab. Association of the kinetics of extracellular vesicles after plasma exchange will be assessed with clinical outcome data.
NCT04298398 Interventional-Clinical Trial (2021) 108 participants	Impact of Group Psychological Interventions on Extracellular Vesicles in People Who Had Cancer	Breast, prostate and colorectal cancer	Perform psychological interventions: Mindfulness-Based Cognitive Therapy (MBCT) and Emotion Focused Therapy for Cancer Recovery (EFT-CR) and explore any effect on extracellular vesicles and on psychological outcomes of people who had cancer.

*: Thyroid gland carcinoma is a cancer of the thyroid, a small gland at the base of the neck that produces hormones. Thyroid nodules are solid or fluid-filled lumps formed within the thyroid, a small proportion of which can be cancerous. *: Prostate cancer, or prostate adenocarcinoma, occurs in the prostate, a gland in the pelvis and part of the male reproductive system.

## Data Availability

Not applicable.
